# Improving Advance Care Planning for People with Parkinson's

**DOI:** 10.1002/mdc3.70706

**Published:** 2026-06-07

**Authors:** Bradley Lonergan, Peter Miller, Elisabete Marques, Yen Tai, Anette Schrag

**Affiliations:** ^1^ Department of Brain Sciences Charing Cross Hospital, Hammersmith, Imperial College London London United Kingdom; ^2^ Partner of PwP with 11 years lived experience; ^3^ Department of Neurology Charing Cross Hospital, Hammersmith, Imperial College Healthcare Trust (ICHT) London United Kingdom; ^4^ Department of Clinical and Movement Neurosciences, UCL Queen Square Institute of Neurology University College London London United Kingdom

**Keywords:** advance care planning, end‐of‐life, multidisciplinary team, palliative care, Parkinson's disease

## Advance Care Planning (ACP) in Parkinson's Disease

As People with Parkinson's (PwP) progress into the advanced and palliative phases, complications (eg, falls, cognitive decline) often lead to a greater reliance on health and social care. Basic palliative care components can be integrated in PD care and tailored to the stage of disease: from the point of diagnosis (eg, emotional support) to end‐of‐life (eg, managing terminal symptoms).^1^ However, Advance Care Planning (ACP) is usually only initiated after these complications develop.

ACP is a shared decision‐making process between patients, their caregivers and clinicians which documents decisions regarding future health and social care utilization. This includes creating a will, appointing a lasting power of attorney, making advance statements (non‐legally binding) and advance decisions to refuse treatment (legally binding; such as “do not attempt cardiopulmonary resuscitation” [DNACPR] forms). If ACP is not in place, surrogates (usually the caregiver) must make decisions on behalf of the patient. However, research has demonstrated that surrogates only correctly predict patients’ treatment preferences correctly 2/3 of the time and they often experience significant regret and psychological sequelae.^2^, ^3^

Given that up to 80% of PwP will have developed dementia after 20 years with PD, the time window during which to make ACP decisions is often limited.^4^ Various national guidelines (eg, UK, Germany and USA) recommend that PwP complete ACP.^5^, ^6^, ^7^ Reported rates of ACP completion in advanced PD around the world vary widely, from 0% to 95%, depending on national (eg, cultural, legal, religious) differences and how ACP completion is measured.^8^, ^9^, ^10^ Barriers to ACP are generalizable across specialties, such as time restraints, lack of adequate resources, clinician reluctance and inadequate training.^8^, ^9^, ^10^, ^11^ ACP barriers specific to PwP are relatively under‐explored but appear to relate to relationships with their healthcare professionals and family members, and cognitive decline.^11^, ^12^

This article explores the current practical issues in implementing ACP for PwP and makes suggestions for how implementation can be improved.

## ACP Awareness amongst PwP

One study in north‐east England found that 76% of PwP had not heard of ACP.^14^ A similar number wanted to know more about ACP, suggesting a large unmet need. Inadequate understanding of ACP has also been shown in PwP in the USA.^11^ Anecdotally, we have found that many PwP wish to discuss ACP and have simply not had an opportunity. Others may need more time to consider ACP after it is first mentioned and return to discuss it at future appointments, having had time to think about their wishes and to consult family members.

Locally, PD‐specialist multidisciplinary teams (MDTs) can normalize and promote ACP awareness on individual (eg, discussions in clinic) and departmental levels (eg, posters in clinic areas). Parkinson's UK and the Michael J. Fox Foundation (MJFF) have useful written information about ACP. The introduction of National Healthcare Decisions Day (held annually on April 16 since 2008, https://theconversationproject.org/nhdd) in the USA and Advance Care Planning Day (held annually since 2024, https://advancecareplanday.org) in the UK demonstrate the growth of awareness and national strategy on ACP. Public discussions and campaigns about ACP with media coverage, particularly those led by PwP and PD charities, may be particularly effective ways to raise ACP awareness. Retired judge Nicholas Mostyn, a high‐profile PwP, has campaigned on the applicability of assisted dying legislation to PwP, but there have been no comparable PwP‐led campaigns on ACP.^15^ PD‐related podcasts (such as Movers & Shakers and 2 Parkies in a Pod in the UK and Substantial Matters, MJFF and Davis Phinney Foundation podcasts in the USA) are very popular with PwP and would be well placed to promote ACP completion amongst PwP.

## Skills and Responsibilities for ACP

The introduction and undertaking of ACP are the shared responsibility of all clinicians involved in a PwP's care, but a lack of resources, time and clear pathways in public healthcare systems can lead to a vacuum of responsibility for ACP.^16^ Palliative care and primary care clinicians are usually best placed to have ACP discussions, but they have limited resources and often lack specialist knowledge on PD. Pressures on clinic time, other disease management priorities (eg, medication titration) and a lack of PD‐specific ACP tools and training lead to ACP often being deprioritized in physician‐led PD clinics.^17^ PD specialist nurses, however, may be well‐placed to initiate ACP discussions, given their holistic skillset. They often form close relationships and maintain strong continuity with PwP; additionally, most PwP prefer to discuss their wishes with PD specialist nurses.^18^ Once initiated, PD physicians should support the ACP process by discussing the risks and benefits of life‐sustaining treatments and completing legal paperwork (eg, DNACPR forms) with PwP.

Most PD specialists want further training in palliative care, suggesting a perceived lack of skills and a willingness to develop them.^19^ The integration of ACP training into specialist nurses’ and physicians’ curricula is therefore desirable. Online ACP training for clinicians, such as that developed for PD in the PD_PAL project (https://www.futurelearn.com/courses/best-care-for-people-with-late-stage-parkinsons-disease-part-one) may be helpful for all PD MDT members.^13^ Local networks and pathways, such as Parkinson's UK (PUK) “*Parkinson's Nurse Learning Pathway*,” can facilitate such ACP training. Greater skills, engagement and confidence amongst PD physicians in palliative care will likely lead to the development of novel approaches, such as trials of home‐based^20^ and holistic MDT‐focused^21^ care, and better overall care for PwP.

## Timing

There is no universally agreed time to initiate ACP discussions. In fact, identifying the optimal timing and referral criteria for PwP is a major barrier to improving access to and integration with palliative care services in general.^22^ In a USA PwP population, 20–25% each preferred to discuss ACP: a) at diagnosis; b) in early PD; c) in advanced PD.^12^ 5% never wanted to discuss ACP, 12% were unsure and 12% wanted clinicians to wait until the PwP asked to discuss ACP. These preferences may differ based on previous healthcare experiences, cultural differences and healthcare availability. A sensitive approach is needed to gauge individual preferences, and we will outline our general approach to ACP timing below.

The peri‐diagnosis period can be an uncertain and anxious time for PwP.^23^ PwP may need time to adapt to the diagnosis and the clinical focus is usually on improving symptoms. Thus, most PwP and clinicians do not wish to discuss ACP at diagnosis.^24^ PwP also often feel they have insufficient time to ask questions at appointments shortly after diagnosis, so clinicians should prioritize answering these questions and building rapport.^25^ Atypical parkinsonism may be an exception to this rule, given that it typically has a delayed diagnosis and a quicker decline.^26^ Explaining ACP within written material given at diagnosis may be a suitable, but potentially sensitive, solution. ACP discussions become more pressing in advanced PD and should certainly be conducted before cognitive impairment develops. Table 1 outlines several approaches that could be used at different times to trigger ACP discussions.

**TABLE 1:** An approach to ACP at different times in the PD journey

Time from diagnosis	ACP approach	Focus
*0–12 months*	*Focus on PwP open to early ACP and those with atypical parkinsonism*	Unlikely to be the preferred time to discuss ACP for most PwP^13^, ^25^ Build rapport and “*therapeutic alliance*” Take a broader view of quality of life and potential care needs (eg, lifestyle, work, finances, living arrangements and driving) More appropriate in atypical parkinsonism, given more rapid progression^27^
*12 months+*	*Raising ACP awareness*	Screening: “*some patients tell us that they want to talk about issues like prognosis or life expectancy up front while others tell us they'd rather wait until later—what do you prefer?*”^13^ Gentle probing during consultations to gauge openness to ACP discussion
*Definition and/or milestone reached*	*Trigger‐led ACP*	Led by PwP; consider when reaching one of the following: Advanced PD (in Med‐ON)^39^ Hoehn & Yahr score 4 or 5 Schwab & England score <50% Decision to eat/drink with acknowledged risk or insert of PEG > 1 unplanned hospital admission in 12 months PD milestone reached (*mean time to mortality*)^40^ Persistent formed visual hallucinations (*5.1 years)* Multiple falls (eg, > 1 fall in 12 months) (*4.1 years)* Early dementia (*3.3 years)* Institutionalization (*3.3 years)* Referral for advanced therapies Aiming to discuss before onset of cognitive symptoms and loss of capacity

PwP should then be proactively given the opportunity to discuss their ACP preferences, at least annually as recommended by the American Academy of Neurology, as they often change over time.^6^, ^10^ Treatment preferences are often unstable and affected by one's experiences of healthcare and disease over time, and it is difficult to predict one's future preferences.^27^ An ongoing ACP dialogue, rather than a single fixed discussion, creates a more accurate shared understanding of a PwP's values.

## PD‐Specific Elements of ACP: Artificial Nutrition and Advanced Therapies

Where ACP is completed by PwP, it is often general and does not address PD‐specific issues.^10^, ^28^ Several adaptations should be made to optimize general ACP for PwP, such as including preferences and clinical eligibility for advanced therapies (eg, apomorphine, Duodopa, foscarbidopa‐foslevodopa and Deep Brain Stimulation [DBS]) as well as artificial nutrition and neuropsychiatric symptom management. The risk–benefit balance for advanced therapies needs to be individualized, eg, where dementia may reduce compliance and increase complications. It may also be appropriate to consider discontinuation of pump therapies, and resumption of oral therapies, if there are safety concerns (eg, due to agitation or behavioral symptoms of dementia) or in a PwP who is actively dying.

Dysphagia is a major cause of morbidity and mortality in advanced PD, affecting almost all patients with advanced PD.^29^ The main treatment decision for advanced dysphagia is whether to pursue artificial feeding (eg, percutaneous endoscopic gastrostomy (PEG) or oral feeding with acknowledged risk, both of which should prompt ACP discussions (see Table 1). A UK (NCEPOD) enquiry into dysphagia in PwP found that ACP completion in this setting is currently inadequate.^30^

International guidelines suggest an individualized approach when considering PEG insertion in PwP.^31^ This differs from the more definitive recommendations for those with motor neuron disease (PEG recommended) and advanced dementia (PEG not recommended).^32^, ^33^ It is therefore desirable that PwP consider their wishes about artificial feeding early and include those wishes in advance decisions.^29^ It should be noted that artificial PEG feeding does not reduce the risk of aspiration pneumonia in PD.^34^ In this context, it is important that ACP discussions explicitly differentiate between long‐term PEG feeding and short‐term nasogastric tube placement for feeding and medication administration during acute illness, as the risk–benefit profiles are clearly different.

## Measuring ACP Outcomes

Measuring the impact of ACP is multifaceted and difficult in real‐world practice. Possible measures include dying at the Preferred Place of Death (PPD), qualitative feedback from former carers, avoiding unplanned hospital admissions and healthcare costs. It is often assumed that most people (including PwP) would choose home as their PPD but, surprisingly, a third of PwP said that a PPD was not important to them.^35^ A Delphi consensus rated a patient receiving “*care consistent with (their) goals*” as the best potential outcome measure for ACP.^36^ However, goal‐consistent care is not measured in a standardized way and goals may change over time. Studies have struggled to demonstrate that ACP has a clearly positive effect on most of its intended outcomes, including on QoL.^37^ However, we suggest that this is due to limitations in measurable ACP outcomes rather than a lack of efficacy. The potential benefits of ACP (protecting PwP autonomy, preparation of the family) are probably better measured with qualitative methodology, particularly during terminal stages with PwP and post‐bereavement with caregivers.

## Documentation

ACP documentation needs to be easy to navigate, change and access for primary care, secondary care and PwP. Our ACP focus group participants expressed frustration at poor communication of their ACP decisions between primary and secondary care, leading to repetitive discussions, and a lack of awareness about existing ACP on admission to hospital.

Electronic ACP is often complicated by the different electronic record systems used within primary and secondary care, which usually run in parallel silos without communicating. An integrated electronic system which provides ubiquitous access, such as the *Universal Care Plan* being developed in London, would significantly improve communication and clarity. This would be improved by giving patients the ability to amend their own preferences, provided there were adequate safeguards in place to protect the vulnerable. Previous attempts, such as *Coordinate My Care* in London, have had limited success, so paper‐based ACP continues to have a role in the short‐term. Across Europe, PwP independently create advance directives much more often than they create electronic ACP with their healthcare providers.^11^

Paper‐based ACP can be patient‐held and stored in a consistent location at home, such as the fridge (eg, Message in a Bottle Scheme in Wales), thus empowering patients with ownership over their decisions. However, it is more likely to be duplicated, lost or poorly communicated between healthcare sites or visits than electronic ACP.

Although various ACP templates already exist, their implementation remains poor in PwP. In the UK, only 1/3 of patients with advanced PD have ACP documented.^38^ Current templates are too lengthy for routine clinical use (eg, PD_PAL) or lack specificity/relevance to PwP (eg, ReSPECT [UK]).^13^, ^37^ Such generic forms could be adapted for PwP by routinely including statements about feeding preferences, but process changes are also required to meaningfully improve implementation rates. For example, PwP would still need adequate education on ACP and different life‐sustaining treatments and healthcare professionals would need better training and more engagement with ACP.

## Approaching ACP in PwP during Clinic

Given the multitude of issues that one needs to cover in short clinic appointments, there is not usually enough time for a full exploration and documentation of ACP. This is probably the biggest barrier to consistent ACP completion in PwP. We suggest that clinicians focus on a few key points in clinic appointments: consider the optimal timing and openness to ACP for each PwP; initiate discussions; and signpost to more resources (see File S1 for more information). For the wider PD service, teams could aim to raise ACP awareness more widely and formalize local/national pathways for ACP completion and documentation.

## Conclusion

Given the growing burden of PD and PwP with complex symptoms, delivering effective palliative care and advocating for ACP represents an increasing challenge for clinicians working with PwP. Whilst we acknowledge the resource limitations in many services, PD services are well‐placed to initiate ACP discussions with PwP. PD clinicians and nurses often maintain continuity with PwP, have developed trust with them over time and have knowledge about the individual's condition.

We have made some suggestions on how PD clinicians and services may approach improving ACP implementation. Future work to improve ACP in PwP needs to take a holistic and integrated approach. This includes encouraging wider recognition of the importance of ACP amongst PwP and PD professionals, improving training and education for PD clinicians, normalizing and routine inclusion of ACP in PD consultations and improving the practical issues (eg, accessibility of documents to PwP and professionals) with ACP implementation.

## Author Roles

(1) Manuscript Preparation: A. Conception; B. Writing of the first draft; C. Review and Critique.

B.L.: 1B, 1C

P.M.: 1C

E.M.: 1C

Y.T.: 1C

A.S.: 1A, 1C

## Disclosures

**Ethical Compliance Statement:** The authors confirm that the approval of an institutional review board was not required for this work. We confirm that informed consent was not required, as this work did not involve any human subjects. We confirm that we have read the Journal's position on issues involved in ethical publication and affirm that this work is consistent with those guidelines.

**Funding Sources and Conflict of Interest:** No specific funding was received for this work. The authors declare that there are no conflicts of interest relevant to this work.

**Financial Disclosures for the Previous 12 Months:** AS has received research funding or support from University College London, National Institute of Health (NIHR), National Institute for Health Research ULCH Biomedical Research Centre, the International Parkinson and Movement Disorder Society (IPMDS), the European Commission and Parkinson's UK. She has received honoraria for consultancy from Biogen, Abbvie, Bial, Otsuka and Lilly. She has received royalties from Oxford University Press.

## Financial Disclosures and Conflicts of Interest

Author disclosures are available in the Supporting Information.

## Supporting information


Data S1.

**Data S2.** COI_disclosure.

## Data Availability

Data sharing not applicable to this article as no datasets were generated or analysed during the current study.
