# Supplementary material for: Improving Advance Care Planning for People with Parkinson's
Source: Mov Disord Clin Pract. 2026 Jun 7:10.1002/mdc3.70706. Online ahead of print. doi: 10.1002/mdc3.70706 (PMC13339008; doi:10.1002/mdc3.70706)
Supplement: Supplementary file 1 — Data S1. [file MDC3-9999-0-s001.docx]

**Recommendations for improving ACP**

1. **In a clinic appointment with a PwP, consider:**
2. *Timing:* consider the optimal timing for ACP discussion for each PwP, according to Table 1. If ACP is not appropriate for someone, either at this appointment or indefinitely, the process ends here.
3. *Openness:* actively gauge openness to discuss ACP, e.g. “Some patients want to know more about what their future with Parkinson’s may look like and to make future plans. Is that something you would like to discuss?”
4. *Discuss:* If open, define and gently discuss the ACP process:
   1. “We call the process of making plans for the future ‘advance care planning’. This is wide-ranging but includes your preferences for certain medical treatments, such as resuscitation, and where you might want to be looked after if you needed more support”
   2. “Do you have a Will/Living Will/Lasting Power of Attorney?”
   3. “Resuscitation is the process of trying to restart the heart, using chest compressions, breathing tubes and medications, after it has stopped. It is only successful ~ 20% if started whilst in hospital and ~ 10% out of hospital. Some people decide that they would not want to be resuscitated if their heart were to stop, because it seems too invasive or they would not have a quality of life that is acceptable to them. You may have heard this called DNAR or ‘do-not-attempt-resuscitation'; is this something you have thought about for yourself?”
5. *Signpost:* for those individuals who would like to know or do more, we recommend signposting them to:
   1. Locally agreed ACP pathway (see below)
   2. PUK documents on advanced PD (in draft)
   3. Compassion in Dying: <https://compassionindying.org.uk/>. PwP can independently create their own ACP documents and store them online
6. **outside of clinic, services could consider the following points:**
7. *Raise awareness of ACP for PwP:* both within your team, supporting teams (e.g. primary care) and with PwP themselves. This could include hosting an ACP awareness event and playing a video or displaying posters in shared areas (e.g. outpatients waiting area).
8. *Create an ACP pathway and/or lead clinician:* consider who is responsible for competing ACP in your service and how referrals will work. This is likely to depend on local factors, but could include primary care physician, PDCNS, palliative care team, geriatrician or neurologist.
9. *Documentation:* considering local services, decide whether ACP forms will be stored physically or electronically.
10. *Share best practice regionally and/or nationally:* this could be extended to specialist PD-specific neuro-palliative care clinics and conferences/events.
